# Incidence of neutropenia and use of granulocyte colony-stimulating factors in multiple myeloma: is current clinical practice adequate?

**DOI:** 10.1007/s00277-017-3191-7

**Published:** 2017-12-27

**Authors:** Xavier Leleu, Francesca Gay, Anne Flament, Kim Allcott, Michel Delforge

**Affiliations:** 1 Department of Haematology and CIC Inserm U1082, Hôpital La Milétrie, Poitiers, France; 2 Myeloma Unit, Division of Haematology, Azienda Ospedaliero-Universitaria, Città della Salute e della Scienza di Torino, Turin, Italy; 3 Amgen (Europe) GmbH, Brussels, Belgium; 4 Oxford PharmaGenesis, Tubney, Oxfordshire, OX13 5QJ UK; 5 Department of Haematology, UZ Leuven, Leuven, Belgium

**Keywords:** Multiple myeloma, Neutropenia, Granulocyte colony-stimulating factors, G-CSF

## Abstract

Although immunomodulatory drugs, alkylating agents, corticosteroids, protease inhibitors, and therapeutic monoclonal antibodies improve multiple myeloma outcomes, treatment burden is still an issue. Neutropenia is a known complication of cytotoxic cancer therapy and is often associated with infections; it is an important consideration in myeloma given the fact that patients often have a weakened immune system. The risk of febrile neutropenia increases with severe and persisting neutropenia. Recombinant granulocyte colony-stimulating factors (G-CSFs) are commonly used to reduce the incidence, duration, and severity of febrile neutropenia. Here, we review the risk and management of neutropenia associated with new and commonly used anti-myeloma agents. Few papers report the use of G-CSF in patients with multiple myeloma receiving anti-cancer treatments, and fewer describe whether G-CSF was beneficial. None of the identified studies reported G-CSF primary prophylaxis. Further studies are warranted to evaluate the need for G-CSF prophylaxis in multiple myeloma. Prophylaxis may be particularly useful in patients at high risk of prolonged severe neutropenia.

**Electronic supplementary material:**

The online version of this article (10.1007/s00277-017-3191-7) contains supplementary material, which is available to authorized users.

## Introduction

Neutropenia is a recognized complication of cytotoxic cancer therapy. Prolonged severe neutropenia increases the risk of serious infections and of febrile neutropenia, which can themselves be life-threatening. In addition, these conditions can lead to dose modifications or delays, which in turn reduce treatment efficacy [1, 2]. As well as having a significant impact on patients’ well-being, febrile neutropenia places a considerable burden on hospital resources, with affected individuals frequently requiring immediate inpatient admission and antibiotic treatment [3].

An understanding of the neutropenia risk associated with multiple myeloma (MM) treatment is particularly important, given the immunodeficiencies caused by the impact of the disease on B cells, T cells, dendritic cells, and natural killer cells [4]. MM is characterized by proliferation of a clonal population of monotypic plasma cells that differentiate from normal B cells within the bone marrow and produce large amounts of immunoglobulin (M-protein), immunoglobulin (Ig) fragments, or light chains [5]. The abnormal expansion of plasma cells disrupts immune homeostasis, which can in turn lead to neutropenia, hypogammaglobulinaemia, and impaired lymphocyte function, all of which increase susceptibility to infection [6]. MM also affects numerous other organs, either directly (e.g. through accumulation of M-protein in the kidneys) or indirectly (e.g. if bone lesions lead to vertebral collapse in the thorax, this can lead to respiratory problems), which predisposes patients to infection [4]. Furthermore, patients with MM are often elderly and can have comorbidities; both factors can be associated with a weakened immune system [7, 8]. In addition, patients frequently receive multiple, and often long, rounds of treatment with regimens that include dexamethasone, which can result in impaired immunity and hyperglycaemia, which in turn increase the risk of infection [4]; indeed, infection is the most frequent cause of death in patients with MM [4].

Antibiotic prophylaxis can prevent infections and reduce mortality in patients receiving cytotoxic therapy [9]; however, most studies of antibiotic efficacy in this setting have been limited to haematological cancers, and the prophylactic use of these agents has raised concerns regarding the development of antibiotic resistance [7, 9]. Therefore, the Infectious Diseases Society of America currently limits its recommendation to the use of quinolones in patients predicted to have prolonged durations of profound neutropenia (absolute neutrophil count [ANC] ≤ 100 cells/mm^3^ after cytotoxic chemotherapy) [10].

Recombinant granulocyte colony-stimulating factor (G-CSF) has been developed to reduce the incidence, duration, and severity of neutropenia and febrile neutropenia by stimulating neutrophil maturation and production in the bone marrow. Studies suggest that patients with MM are at high risk of infections during the first 2 months of chemotherapy [11]. Although G-CSF administration will not be effective in reducing the risk of infection if the cause of neutropenia is independent of the G-CSF pathway, or if the mechanism by which the drug increases the risk of infections is not related to neutropenia (such as in the case of dexamethasone), appropriate use of G-CSF prophylaxis can be particularly important to reduce the risk related to a low neutrophil count.

Given the high risk of infection in patients with MM, the immune toxicity of many of the agents used to treat the disease and the various mechanisms by which these agents reduce neutrophil count, we wished to gain a better understanding of the incidence of neutropenia in the treatment of MM and the use of G-CSF prophylaxis. Therefore, we reviewed the literature, with a focus on common standard regimens and important new agents.

### Neutropenia associated with commonly used regimens in MM

To assess the risk and management of neutropenia associated with new and commonly used anti-myeloma agents, we searched the literature (titles, abstracts, and keywords) in February 2016 for phase 3 clinical trials or observational studies that reported neutropenia-related outcomes in patients with MM. We did not consider studies that were investigating the efficacy of stem cell transplantation (SCT) because for these studies, the main reported G-CSF-related outcomes were mobilization and neutrophil engraftment. Key studies on the newest regimens that were published after February 2016 were selected by authors for inclusion.

Although grade 3–4 neutropenia is a recognized complication of lenalidomide and pomalidomide treatment [12, 13], our literature search revealed that it is widespread in patients with MM, with an incidence of over 10% reported for 28 different regimens (Table 1). Neutropenia rates reported below reflect the incidence of grade 3 or 4 events.

**Table 1:** Neutropenia rates by regimen

Regimen	Range of reported grade 3 and 4 neutropenia, %	Number of studies
Thalidomide	0–≤ 5	1
CTD	0–≤ 5	1
VT	0–≤ 5	1
VP	0–≤ 5	1
Bortezomib	0–≤ 5	2
	> 5–≤ 10	1
	> 10–≤ 25	7
	> 25–≤ 50	0
VD	0–≤ 5	2
	> 5–≤ 10	1
	> 10–≤ 25	2
VTD	0–≤ 5	2
	> 10–≤ 25	2
	> 25–≤ 50	1
Bortezomib-based	> 5–≤ 10	1
	> 10–≤ 25	2
	> 25–≤ 50	1
Dexamethasone	0–≤ 5	5
	> 10–≤ 25	1
Lenalidomide maintenance	> 5–≤ 10	2
	> 10–≤ 25	2
	> 25–≤ 50	1
RD	0–≤ 5	1
	> 5–≤ 10	1
	> 10–≤ 25	11
	> 25–≤ 50	9
	> 50–≤ 75	2
RP maintenance	> 5–≤ 10	1
RVD	> 5–≤ 10	1
	> 10–≤ 25	1
Lenalidomide-based	> 10–≤ 25	2
	> 25–≤ 50	5
ERD	> 25–≤ 50	1
MP	> 5–≤ 10	1
	> 25–≤ 50	2
MPT	> 10–≤ 25	1
	> 25–≤ 50	1
PAN-BTZ-Dex	> 25–≤ 50	1
VCD	> 10–≤ 25	1
	> 25–≤ 50	2
VMP	> 10–≤ 25	1
	> 25–≤ 50	2
VMPT	> 25–≤ 50	1
PAD	> 10–≤ 25	2
RCD consolidation	> 10–≤ 25	1
PD	> 25–≤ 50	4
PVD	> 25–≤ 50	1
IRD	> 10–≤ 25	1
Vorinostat + bortezomib	> 10–≤ 25	1
KRD	> 25–≤ 50	1
MPR	> 50–≤ 75	1
	> 75–100	1
CHOP	> 50–≤ 75	1
HD-M	> 50–≤ 75	1
	> 75–100	3
VTD-PACE	> 50–≤ 75	1
	> 75–100	1
PACE	> 75–100	1

*CHOP* cyclophosphamide/doxorubicin/vincristine/prednisone, *CTD* cyclophosphamide/thalidomide/dexamethasone, *ERD* elotuzumab/lenalidomide/dexamethasone, *HD-M* high-dose melphalan, *IRD* ixazomib/lenalidomide/dexamethasone, *KRD* carfilzomib/lenalidomide/dexamethasone, *MP* melphalan/prednisone, *MPR* melphalan/prednisone/lenalidomide, *MPT* melphalan/prednisone/thalidomide, *PACE* cisplatin/doxorubicin/cyclophosphamide/etoposide, *PAD* bortezomib/doxorubicin/dexamethasone, *PD* pomalidomide/dexamethasone, *PVD* pomalidomide/bortezomib/dexamethasone, *PAN-BTZ-Dex* panobinostat/bortezomib/dexamethasone, *RCD* lenalidomide/cyclophosphamide/dexamethasone, *RD* lenalidomide/dexamethasone, *RCP* lenalidomide/cyclophosphamide/prednisone, *RP* lenalidomide/prednisone, *RVD* lenalidomide/bortezomib/dexamethasone, *VCD* bortezomib/cyclophosphamide/dexamethasone, *VD* bortezomib/dexamethasone, *VMP* bortezomib/melphalan/prednisone, *VMPT* bortezomib/melphalan/prednisone/thalidomide, *VP* bortezomib/prednisone, *VT* bortezomib/thalidomide, *VTD* bortezomib/thalidomide/dexamethasone, *VTD-PACE* bortezomib/thalidomide/dexamethasone/cisplatin/doxorubicin/cyclophosphamide/etoposide

## Neutropenia with current anti-myeloma regimens

### Bortezomib–dexamethasone-based regimens

A bortezomib–dexamethasone (VD) backbone is still widely used at first line for transplant-eligible patients; however, we found relatively few reports of neutropenia with VD-based triplet combinations. Neutropenia rates of 25% or lower were reported in two observational studies and a phase 3 study of patients receiving lenalidomide with VD; similar neutropenia rates were reported in the majority of studies investigating the addition of doxorubicin to VD (all phase 3 clinical trials) and the majority of phase 3 and observational studies in which thalidomide was added to VD [14–22]. In contrast, two of three phase 3 studies of combinations of cyclophosphamide and VD reported that neutropenia affected 40% of patients; neutropenia was also reported in 50% of participants in an observational study of the same regimen [17, 21, 23]. Only two studies reported G-CSF use in patients receiving a VD-based triplet regimen. In the retrospective observational study of lenalidomide with VD by Jimenez-Zepeda et al., 8 of 30 (3.75%) patients were administered G-CSF, and in a clinical trial by Palumbo et al. investigating doxorubicin with VD, 15 of 64 patients (4.3%) received G-CSF [15, 22].

### Immunomodulatory drug-based regimens

**Fig. 1:**
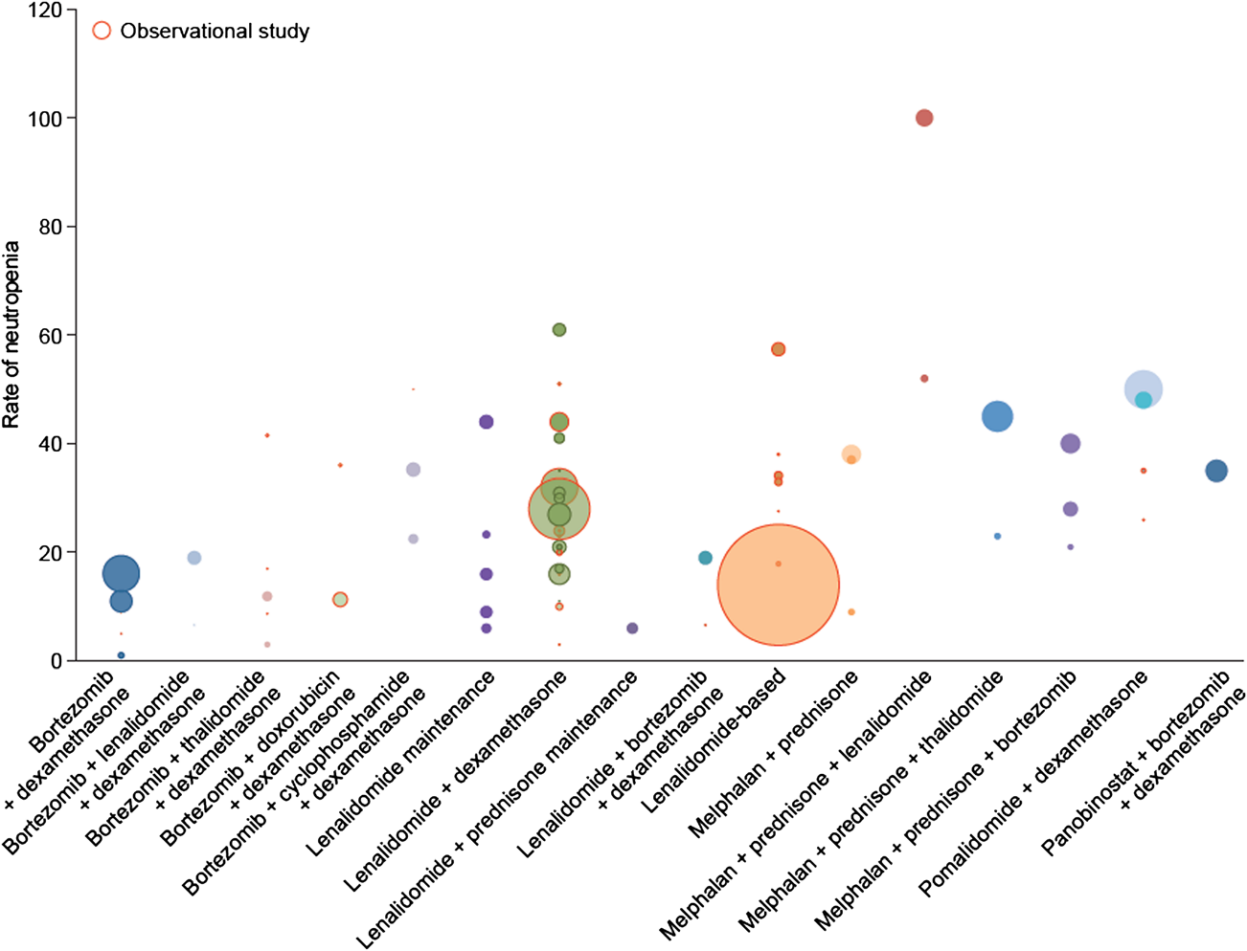
Rates of grade 3 and 4 neutropenia in commonly used regimens for the treatment of multiple myeloma. Each bubble represents one study. The size of the bubbles represents the number of patients in the study. Bubbles outlined in red are observational studies; the remainder are phase 3 clinical trials. Two points for lenalidomide + dexamethasone are not visible: an observational study with a neutropenia incidence of 16% (*N* = 50) and an observational study with a neutropenia incidence of 35% (*N* = 31)

Lenalidomide–dexamethasone (RD)-containing regimens are among the most commonly used in patients with MM but are also among those with the highest reported incidences of neutropenia (Fig. 1). Lenalidomide can be combined with low- or high-dose dexamethasone; for simplicity, we included RD-based regimens regardless of the dose of dexamethasone used. Although RD was initially reported as low risk based on the pivotal phase 3 trial [8, 24], we found other phase 3 and observational studies reporting higher incidences of neutropenia with RD (up to 61%) (Fig. 1) [14, 25–46]. Infection rates were moderate compared with neutropenia rates (Table 2), reaching 37% in the expanded access study by Sun et al. [45]; however, rates of febrile neutropenia were low (< 1–6%, Table 2).

**Table 2:** Infection rates in regimens associated with high levels of neutropenia in selected regimens of interest

Author/date	Regimen	Grade 3 and 4 neutropenia, %	Grade 3 and 4 infection, %	Febrile neutropenia, %
Bortezomib–dexamethasone-based				
Jimenez-Zepeda et al. [15]	RVD	7	26	NR
Durie et al. [14]	RVD	19	NR	NR
Garcia-Sanchez et al. [16]	VTD	9	NR	NR
Takashima et al. [19]	VTD	17	21 (not specified if grade 3–4)	NR
Wu et al. [20]	VTD	43.3 (VTD) 40 (improved VTD)	NR	NR
Moreau et al. [17]	VTD	12	NR	NR
Niesvizky et al. [18]	VTD	3	NR	NR
Palumbo et al. [22]	PAD	36	15 (serious infection)	3
Mai et al. [21]	PAD	11	13 (serious infection)	NR
Moreau et al. [17]	VCD	23	NR	NR
Mai et al. [21]	VCD	35	11 (serious infection)	NR
Kusano et al. [23]	VCD	50	NR	NR
Melphalan–prednisone-based				
Hulin et al. [47]	MP	9	NR	NR
Richardson et al. [48]	MP	38	NR	4
Palumbo et al. [49]	MP	37	7	0
Palumbo et al. [50]	MPR	52	0.8	NR
Palumbo et al. [49]	MPR plus lenalidomide maintenance	67 grade 3 and 35 grade 4	11	7
	MPR	64 grade 3 and 32 grade 4	15	2
Hulin et al. [47]	MPT	23	Neutropenia did not translate into more frequent severe infections	
Facon et al. [28]	MPT	45	17	3
Niesvizky et al. [18]	VMP	21	NR	NR
Richardson et al. [48]	VMP	40	NR	3
Palumbo et al. [51]	VMP	28	9	2
Lenalidomide–dexamethasone-based				
Dimopoulos et al. [27]	RD (low dose)	3	NR	NR
Dimopoulos et al. [27]	RD (intermediate dose)	23	NR	NR
Mookerjee et al. [35]	RD	11	NR	NR
Firatli Tuglular et al. [30]	RD	10	NR	NR
Durie [14]	RD	21	NR	NR
Zonder et al. [39]	RD	21	16	NR
Beksac et al. [25]	RD	16	NR	4
Tosi et al. [38]	RD	35	15	NR
Fouquet et al. [32]	RD	16	NR	NR
Family et al. [29]	RD (4 cycles)	24		3.8
Huang et al. [34]	RD	20	9	NR
Dimopoulos et al. [26]	RD	17		0
Firatli Tuglular et al. [31]	RD	24	11	NR
Gay et al. [52]	RD	24	6	NR
Moreau et al. [36]	RD	16	NR	NR
Weber et al. [46]	RD	41	22	3
Dimopoulos et al. [41]	RD	30	11	3
Geraldes et al. [33]	RD	35	25	NR
Schwarzer et al. [37]	RD	32	11	<1
Facon et al. [28]	RD	28	29	1
Leleu et al. [42]	RD	31	NR	3
Stewart et al. [44]	RD	27	NR	NR
Lonial et al. [43]	RD	44	NR	NR
Sun et al. [45]	RD	61	37	NR
Alegre et al. [40]	RD	51	NR	6
Durie et al. [14]	RVD RD	19 21	NR	NR
Jimenez-Zepeda et al. [15]	RVD	7	26	NR
Pomalidomide-based				
Dimopoulos et al. [53]	PD	50	NR	NR
Maciocia et al. [54]	PD	35	Neutropenic sepsis, 11	NR
Miles and Wells [55]	PD	26	Sepsis requiring admission, 24	NR
San Miguel et al. [56]	PD	48	30	10
High-dose melphalan				
Cook et al. [57]	HD-M plus salvage ASCT	75	NR	NR
Palumbo et al. [58]	HD-M	77	40	17
Palumbo et al. [50]	HD-M	94	16.3 (includes febrile neutropenia)	NR
Gay et al. [59]	HD-M	80	19	NR

*ASCT* autologous stem cell transplantation, *HD-M* high-dose melphalan, *MP* melphalan/prednisone, *MPR* melphalan/prednisone/lenalidomide, *MPT* melphalan/prednisone/thalidomide, *NR* not reported, *PD* pomalidomide/dexamethasone, *RD* lenalidomide/dexamethasone, *RVD* lenalidomide/bortezomib/dexamethasone, *VMP* bortezomib/melphalan/prednisone

When reported, G-CSF use was between 22 and 54% and was initiated after the manifestation of neutropenia. In a phase 3 trial by Dimopoulos et al., G-CSF was used to manage neutropenia in 22% of patients. In the same study, the neutropenia rate was 30%, the infection rate was 11%, and dose reductions or interruptions due to any adverse event (AE) were recorded in 76% of patients [41]. In a similar phase 3 study by Weber et al., in which 41% of patients experienced neutropenia and 22% had an infection, 34% received reactive G-CSF and dose reductions were required in 77% of participants (neutropenia was mentioned as a primary reason for dose reduction) [46]. In the observational study by Leleu et al., 31% of patients experienced neutropenia and 23% received G-CSF, which the authors noted was probably reactive. Dose reductions and interruptions with neutropenia as the primary cause were rare [42]. In the retrospective analysis of patients on the MM-016 extended access programme by Sun et al., intermittent G-CSF (4–6 doses per cycle) was used reactively in 49% of patients and as secondary prophylaxis in 5%. Infection rates remained high, but patients receiving G-CSF were able to receive RD for longer than those who did not receive G-CSF, which appeared to lead to improved response rates [45]. In the French observational study by Fouquet et al., 34% of patients had a dose reduction owing to either neutropenia or thrombocytopenia [32]; in the Greek study by Katodritou et al., neutropenia was the most common AE leading to treatment discontinuation (7%) [60]. G-CSF use was not reported in either publication.

Pomalidomide with dexamethasone has been approved for patients who have received at least two previous therapies including bortezomib and lenalidomide [61]. Phase 3 studies suggest that the risk of grade 3 or 4 neutropenia (approximately 50%) is slightly higher with pomalidomide than with lenalidomide (Fig. 1) [53, 56]. Subsequent analysis of one study found that neutropenia was the most common AE leading to dose reductions and interruptions (4.7 and 19.4% of patients, respectively) [62]. Although San Miguel et al. noted that neutropenia did not necessarily translate into infection, the data showed that in the active arm, 30% of patients experienced infection (compared with 24% in the control arm) and 10% experienced grade 3 or 4 febrile neutropenia, which was much higher than the incidence in the control arm (< 1%) [56].

It should be noted that pomalidomide has so far been studied in heavily pre-treated patients who are therefore likely to have advanced disease and reduced bone marrow function; in the pivotal phase 3 study, the median number of previous treatments was five, and over 90% of patients had received more than two previous treatments [56]. Neutropenia rates in patients using pomalidomide at earlier lines of therapy may therefore be lower than the rates reported in the phase 3 study. Two small observational studies suggest that the real-world incidence of neutropenia with this regimen may be slightly lower. However, despite the lower rates of neutropenia, in the observational study by Maciocia et al., 11% of patients had grade 3 or 4 neutropenic sepsis and Miles et al. reported sepsis requiring hospital admission in 24% of patients [54, 55]. Additional data in a larger population would be needed to confirm these findings.

In common with lenalidomide, the phase 3 studies reported that dose interruptions and dose reductions with pomalidomide were common (67 and 66%, and 27 and 22% for interruptions and reductions, respectively, in the two studies) [53, 56]. G-CSF use was reported only by San Miguel et al. and was used in 43% of patients [56].

### Melphalan–prednisone-based regimens

All studies of melphalan–prednisone-based regimens were clinical trials; we did not find any observational studies reporting neutropenia rates. Melphalan and prednisone (MP) alone were associated with a risk of neutropenia of less than 10% in one trial and a higher risk (37–38%) in two trials (Table 2) [47–49]. However, the combination of melphalan, prednisone, and lenalidomide (MPR) was consistently associated with neutropenia in over half of patients [49, 50]. In contrast, although combining melphalan and prednisone with thalidomide (MPT) or bortezomib (VMP) was also associated with neutropenia incidences of over 20%, there were no reports of these agents causing neutropenia in more than 50% of patients [18, 28, 47, 48, 51].These differences are illustrated by the recently published head-to-head study of MPR versus MPT, which found that the incidence of neutropenia with MPR was more than twice that with MPT [63]. This indicates that the risk of neutropenia is affected by the type and the number of agents in a regimen, and highlights the importance of considering strategies to reduce the neutropenic risk.

The patient population should also be considered when interpreting these data: MP-based combinations are usually recommended for patients not eligible for SCT. Such patients are typically elderly with poor performance status, both of which are recognized risk factors for neutropenia [7]. Indeed, all five studies of VMP and MPT were in patients who were elderly or not eligible for transplantation or high-dose therapy. Whereas a study of MPR in transplant-eligible patients reported a neutropenia rate of 52% [50], neutropenia rates were approximately 100% (64–67% grade 3 and 32–35% grade 4) in the study of transplant-ineligible patients [49]. In clinical practice, VMP is the most commonly used melphalan-containing regimen and MPR is not currently recommended by some guidelines [64].

Despite the high rates of neutropenia seen with these triplets, few patients experienced infections, and rates of febrile neutropenia were low (Table 2). Notably, Hulin et al. found that in their trial of MPT in elderly patients, neutropenia did not appear to result in more frequent serious infection [47]. This is particularly encouraging, given that infection can be more serious in elderly patients than in younger individuals. The low rate of infection compared with RD regimens could reflect the tolerability profile of prednisone, which differs from that of dexamethasone. Moreover, real-world infection rates may differ from those seen in these phase 3 studies; the selection criteria for clinical trials might exclude the frailest patients who could be most likely to experience infections.

Hulin et al. reported that thalidomide increased the requirement for dose reductions (for any reason) owing to AEs when added to MP (20 vs 3%) [47]. Richardson et al. was the only paper describing an MP study that reported the number of dose reductions due to neutropenia. In this study, bortezomib and melphalan were reduced in 2 and 5% of patients, respectively, in the VMP arm and melphalan was reduced in 7% of patients in the MP arm [48]. In the same study, G-CSF was used in 21 and 23% of patients, respectively [48]. However, the publication did not specify whether G-CSF was used prophylactically or reactively, so it is difficult to draw any conclusions regarding the relationship between its use and rates of neutropenia and infection or dose maintenance.

## Neutropenia associated with newer treatment regimens

In the past 2 years, several new therapies have been approved for the treatment of patients with MM. Panobinostat in combination with bortezomib and dexamethasone has been approved for patients with relapsing and/or refractory multiple myeloma (RRMM) who have received at least two previous regimens including bortezomib and an immunomodulatory agent [65]. In the phase 3 study, neutropenia was reported in 35% of patients. However, the evidence base for this agent is still small [66]. Carfilzomib has recently been approved in combination with lenalidomide and dexamethasone, and with dexamethasone alone, for patients with RRMM [67]. Neutropenia of grade 3 or higher has been reported in the triplet combination [44], but this is likely to reflect the AE profile of lenalidomide, rather than that of carfilzomib (the incidence was 30% with carfilzomib vs 27% with lenalidomide and dexamethasone). In a double-blind, placebo-controlled phase 3 study conducted to assess progression-free survival in patients with MM receiving lenalidomide in combination with dexamethasone and ixazomib, neutropenia was observed in 23% of patients receiving active treatment and 24% of patients receiving placebo [68]. In a phase 3 study comparing lenalidomide with dexamethasone and daratumumab with lenalidomide and dexamethasone alone, neutropenia occurred more frequently in the daratumumab group (52%) than in the control group (37%). Corresponding figures for febrile neutropenia were 5.7% (all of grade 3 or 4) and 2.5% (all of grade 3 or 4), respectively [69]. The incidence of neutropenia was lower in patients treated with VD–daratumumab than in those receiving VD alone in a phase 3 randomized trial: 12.8 and 4.2%, respectively [70]. In another phase 3 trial comparing elotozumab plus lenalidomide and dexamethasone with lenalidomide plus dexamethasone alone (control group), neutropenia occurred at a lower rate in the elotozumab group (34%) than in the control group (44%) [71].

## Regimens associated with a very high incidence of neutropenia

We found several regimens associated with a very high incidence (76–100%) of neutropenia. High-dose melphalan (HD-M) as part of SCT is a standard treatment approach in transplant-eligible patients and was reported to be associated with neutropenia in five publications [50, 57–59]. Infection rates were high in the report of the phase 3 trial by Palumbo et al., despite prophylactic G-CSF administration (40% of patients had an infection and 17% had febrile neutropenia) [58]; however, considering the high rates of neutropenia, infection rates were relatively low in the other studies (Table 2), such as in Palumbo et al.’s report of their HD-M trial (16%) [50]. However, in both studies, patients were younger than 65 years of age; the risk–benefit profile of this regimen is likely to be different in elderly patients. Despite the high toxicity associated with this treatment approach, SCT remains the optimal treatment for patients with early-stage disease [59].

Other regimens associated with very high levels of neutropenia were bortezomib, thalidomide, dexamethasone and cisplatin, doxorubicin, cyclophosphamide and etoposide (VTD-PACE) in patients with NDMM, and cisplatin, doxorubicin, cyclophosphamide and etoposide (PACE) in patients with RRMM, which resulted in neutropenia rates of 79 and 83% and febrile neutropenia rates of 26 and 33%, respectively [72, 73].

## Pegfilgrastim in MM

Over two decades ago, filgrastim, the first daily G-CSF, was approved for reducing the risk of febrile neutropenia in patients receiving cytotoxic chemotherapy for cancer [74]. Since then, lenograstim has also been developed as a daily G-CSF [75]. These agents are also indicated for mobilizing peripheral blood progenitor cells and for reducing the duration of neutropenia in patients undergoing bone marrow transplantation [74, 75]. In 2002, the first long-acting G-CSF, pegfilgrastim, was approved in Europe for reducing the duration of neutropenia and the incidence of febrile neutropenia in adults receiving cytotoxic chemotherapy for cancer [76]. Pegfilgrastim may be preferred over filgrastim by patients and physicians owing to its reduced clearance, which means that only one dose is required per chemotherapy cycle [76]. Pegfilgrastim has also been shown to be more effective than filgrastim at reducing neutropenia and febrile neutropenia in clinical practice [77, 78]. More recently (in 2013), lipegfilgrastim, another long-acting G-CSF, became available for febrile neutropenia prophylaxis [79].

To understand whether long-acting G-CSFs are used in the management of neutropenia in patients with MM, we performed an additional search for articles reporting pegfilgrastim use in patients with this disease. Our search was limited to articles published between 1 January 2013 and 17 February 2016 to capture current clinical practice. Few studies published in the past 3 years reported using pegfilgrastim in MM (*N* = 16) (Supplementary Table 1).

## Pegfilgrastim for neutropenia prophylaxis

Most studies investigated the use of pegfilgrastim with SCT to promote neutrophil engraftment; however, we found five reports of pegfilgrastim being used for neutropenia prophylaxis in patients with MM. Two of these studies used pegfilgrastim in lenalidomide-based regimens. An observational study of patients with RRMM found that pegfilgrastim was given to 8% of patients receiving RD [42]. Daily G-CSF was used more frequently (16% of patients). The authors stated that G-CSF use was likely to have been reactive, not prophylactic.

Pegfilgrastim was used as primary prophylaxis in a dose-escalation study of lenalidomide, doxorubicin, and dexamethasone for patients with RRMM [80]. Pegfilgrastim allowed the dose of lenalidomide to be raised from 15 to 25 mg, in combination with doxorubicin 9 mg/m^2^ and dexamethasone 40 mg. At this final dose, the maximum tolerated dose threshold of 33% or higher incidence of dose-limiting toxicity was still not reached; 48% of patients experienced grade 3 or 4 neutropenia and 23% required dose reductions. The increased dose of lenalidomide facilitated by the addition of pegfilgrastim allowed a much larger proportion of patients to achieve a very good partial response or complete response, compared with the lower dose level (74 vs 23%).

Pegfilgrastim primary prophylaxis has been reported in a phase 1 study of hydroxychloroquine with cyclophosphamide, dexamethasone, and rapamycin in patients with RRMM. Of 15 patients evaluated for toxicity, one experienced grade 4 neutropenia [81]. One retrospective study compared pegfilgrastim and filgrastim primary prophylaxis for reducing neutropenia following SCT. The analysis found no statistically significant differences in median time to leukocyte recovery or duration of febrile neutropenia [82]. The authors concluded that the main difference between filgrastim and its pegylated form is the more convenient formulation of pegfilgrastim as a single fixed dose than as multiple daily administrations.

A prospective cohort study in which patients with advanced MM were treated with reactive filgrastim during their first course of chemotherapy and with pegfilgrastim prophylaxis during their second course reported that pegfilgrastim appeared to reduce the incidence of neutropenia to a greater extent than did daily injections of filgrastim [83].

## Tolerability of pegfilgrastim

Looking across all studies of pegfilgrastim, the agent appeared to be generally well tolerated. The prospective cohort study of sequential filgrastim and pegfilgrastim treatment reported that the main AEs following pegfilgrastim administration were mild fever and bone pain, which were experienced by 12% of patients [83]. In the observational IMPACT study, no adverse drug reactions to any G-CSF were reported [42]. Another study assessing pegfilgrastim with and without cyclophosphamide reported no hospitalizations due to toxicity in the pegfilgrastim group [84]. When pegfilgrastim was administered 6 days after a chemotherapy regimen of lenalidomide, doxorubicin, and dexamethasone, no treatment-related mortality was reported, and rates of febrile neutropenia and venous thromboembolism were less than 10% [85–87]. One phase 1 study assessed escalating doses of melphalan and carfilzomib with a constant pegfilgrastim dose. Although efficacy and safety data are not specific to pegfilgrastim, the authors did report infection in 58% of patients, febrile neutropenia in 33%, and pneumonia, bacteraemia and urinary tract infection each in 8% of patients [88]. A similar phase 1 dose-escalation study reported four episodes of thrombocytopenia, one episode of neutropenia and five episodes of lymphopenia, all grade 4 in severity, in a population of 15 patients [81]. Although these data were more specific for the chemotherapy treatment of hydroxylchloroquine (at ascending doses) along with cyclophosphamide and rapamycin, patients were also treated with pegfilgrastim on day 6.

## Characteristics of patients receiving pegfilgrastim

In the observational study by Leleu et al. of patients with RRMM receiving RD, those who received pegfilgrastim were more likely to have International Staging System stage III at diagnosis, more than four previous treatments and more comorbidities than those who were given daily G-CSFs [42]. They were also more likely to be younger and to be receiving the recommended 25 mg dose of lenalidomide [42]. This suggests that pegfilgrastim may be reserved for patients with a high risk of neutropenia. Aside from that publication, we did not find any reports of the baseline characteristics of patients with MM who were given pegfilgrastim.

However, studies reporting pegfilgrastim use in patients with various haematological malignancies including MM, but that do not report MM data separately, do give details of the baseline characteristics of patients prescribed pegfilgrastim in real-world practice. Several single-centre studies comparing patients who received daily G-CSF with those receiving pegfilgrastim for reducing time to neutrophil engraftment found no differences between the two groups in terms of the baseline characteristics of the patients, although non-significant differences in patient age were seen [89–91]. In Carlino et al., patients receiving pegfilgrastim had a median age of 51 years, compared with 62 years in those receiving daily G-CSFs. Herbert et al. reported median ages of 50 and 56 years for those receiving pegfilgrastim and filgrastim, respectively. Another single-centre study comparing these agents for neutrophil engraftment found that patients given pegfilgrastim were younger than those receiving filgrastim (median age 46 vs 54 years; *P* = 0.05) [92]. This pattern of treatment could be due to a tendency to treat younger patients with more aggressive or intensive treatment than older patients.

## Implications for clinical practice

Together, the studies described above indicate that G-CSF is not regularly used prophylactically in patients with MM. The European Organisation for Research and Treatment of Cancer recommends G-CSF prophylaxis for patients undergoing a chemotherapy regimen with a high (≥ 20%) risk of febrile neutropenia and for patients receiving a chemotherapy regimen with an intermediate (10–20%) risk of febrile neutropenia if they have additional risk factors [7]. In patients with MM, it has been suggested that G-CSF prophylaxis should be administered to those who are undergoing treatment regimens associated with a neutropenia rate of over 50% (those that combine lenalidomide with doxorubicin and dexamethasone, with MP or with cyclophosphamide and dexamethasone). G-CSF prophylaxis is also advised for patients with MM who have additional risk factors (e.g. those aged > 65 years, frail patients and patients with comorbidities) who are receiving a regimen associated with an intermediate risk of neutropenia (e.g. triplet regimens containing bortezomib) [8]. It may also be prudent to consider G-CSF use in patients with aggressive disease to help to avoid the need for treatment delays that may otherwise be required if neutropenia occurs [3]. This can be of particular importance during the first cycles of treatment (at diagnosis and at relapse), when the risk of cytopenia and of infections related to the high tumour burden can be higher. In the relapse setting, the risk of infections may also be particularly high (see pomalidomide data in heavily pre-treated patients), and considering prophylaxis for neutropenia in such patients may be prudent, to remove at least the risk of infections related to the low neutrophilic count. G-CSF is also given reactively in patients who develop neutropenia.

Reports of the use of pegfilgrastim in patients with MM are rare. Although the use of a long-acting G-CSF has been shown to improve outcomes versus the use of daily G-CSFs in other tumour types [77, 78], and there are suggestions that the use of pegfilgrastim could lead to improved outcomes for patients with MM [80], the body of evidence is too small to draw firm conclusions. Large retrospective studies or prospective clinical trials will be needed to determine whether there is an advantage of using pegfilgrastim in this patient population.

It is also important to consider the mechanisms by which MM treatments cause neutropenia. Cytotoxic chemotherapies, such as the alkylating agent melphalan [93], target rapidly proliferating cells and therefore kill myeloid cells as well as malignant cells. However, agents with different mechanisms of action, such as immunomodulatory drugs and monoclonal antibodies, also induce neutropenia. Lenalidomide-induced neutropenia is thought to be associated with the loss of the transcription factor PU.1, which is required for granulopoiesis and neutrophil maturation [20]. The monoclonal antibody daratumumab has recently been approved for use as a monotherapy in Europe and as a monotherapy and in combination with RD or VD in the USA. Neutropenia has been reported for all regimens, but particularly when daratumumab is combined with RD. The mechanism is unclear, but the target of daratumumab, CD38, is known to regulate neutrophil chemotaxis and is present on myeloid stem cells [94, 95]. Neutropenia has also been associated with the monoclonal antibody rituximab; in this setting, it has a late onset and is thought to correlate with B cell recovery following treatment. Rapidly expanding B cells consume the chemokine stromal cell-derived factor 1, which is required for neutrophil egress from the bone marrow [96]. This illustrates the variety of mechanisms by which drugs can induce neutropenia.

As noted above, few papers described G-CSF use in studies of anti-cancer agents in MM, and fewer still evaluated whether or not G-CSF use had a beneficial effect. Further research is required in order to understand how to optimize G-CSF prophylaxis according to the anti-cancer agent used.

## Discussion and conclusions

The pathophysiology of MM, and its tendency to occur in elderly patients, means that neutropenia (and potentially infection) is a common occurrence. MM is a long-lasting disease that requires several treatment courses. Patients may have pre-existing hypogammaglobulinaemia and lymphopenia, in addition to neutrophil destruction by anti-cancer agents. Physicians need to be aware of the incidence and causes of low neutrophil counts, in addition to other risk factors for infection, in order to understand the risk for each of their patients and to manage this risk effectively.

Although infections and febrile neutropenia are lower than may be expected, we found a lack of data on antibiotic prophylaxis in the identified studies, so it is unclear to what extent antibiotics may be influencing infection rates. One of the most important factors influencing infection risk is the duration of neutropenia. This is generally short with most of the commonly used outpatient regimens, which may explain the low incidence of infections. Much of the available data are, however, from clinical trials, in which the patients represent a highly selected population with fewer comorbidities than the overall population. Therefore, rates of infection in clinical practice may be expected to be higher than those reported in the trials, particularly in patients who are elderly, in those with comorbidities, those who have very aggressive disease or those who have received multiple lines of therapy. In these patients, preventing neutropenia is particularly important and may help to avoid treatment delays or dose reductions, thus maximizing patient benefit.

Despite the importance of reducing the incidence and duration of neutropenia, few studies report using G-CSF in this patient population, and none of the studies we identified reported primary prophylaxis with G-CSF. Similar inconsistencies in reporting were found by Chan et al. in their analysis of the reporting of supportive care use in clinical trials published between January 2005 and June 2009 [97]. It is therefore difficult to draw conclusions about the potential role of G-CSF in MM. Nevertheless, two studies did report that G-CSF use permitted increased durations of chemotherapy use, which translated into improved response rates.

Further studies are warranted to evaluate the need for G-CSF prophylaxis in MM. If such treatment is required, there are preliminary suggestions that pegfilgrastim may be preferred over daily G-CSFs owing to the single-use formula. Pegfilgrastim may be particularly useful in patients at high risk of prolonged severe neutropenia, owing to the myelotoxicity of the regimen; patient risk factors such as old age, frailty or poor compliance; disease risk factors such as high tumour burden or a combination of all three.

## Electronic supplementary material

ESM 1 (DOCX 73.9 KB)
