# Supplementary material for: Incidence of neutropenia and use of granulocyte colony-stimulating factors in multiple myeloma: is current clinical practice adequate?
Source: Ann Hematol. 2017 Dec 27;97(3):387–400. doi: 10.1007/s00277-017-3191-7 (PMC5797221; doi:10.1007/s00277-017-3191-7)
Supplement: Supplementary file 1 — (DOCX 73.9 KB) [file 277_2017_3191_MOESM1_ESM.docx]

**Supplementary Table 1** Summary of efficacy and safety data following pegfilgrastim treatment in MM populations

| Reference | Pegfilgrastim regimen | Study design, patient group | *N* | Chemotherapy regimen/ treatment | Pegfilgrastim-related efficacy | Pegfilgrastim-related safety | Recommendations |
| --- | --- | --- | --- | --- | --- | --- | --- |
| **Studies of MM populations** | | | | | | | |
| Neutropenia prophylaxis | | | | | | | |
| Cerchione et al. 2015 [1]  Primary prophylaxis | Pegfilgrastim 6 mg s.c. day +3 | Prospective cohort  RRMM | 41 | Filgrastim  Pegfilgrastim  (regimens included bortezomib, lenalidomide, bendamustine, melphalan and/or doxorubicin) | During pegfilgrastim treatment, neutropenia duration was never >8 days, with a consequent reduction in neutropenia-related infections  During pegfilgrastim prophylaxis, neutropenia was shorter than during filgrastim treatment (median, 4 days [3–7]) | Pegfilgrastim was well tolerated in all patients; the main side effects were mild fever and bone pain in 95/41 patients (12%) | In patients affected by MM exposed to myelosuppressive agents in advanced phases of MM, pegfilgrastim seems to reduce the incidence of neutropenia and may increase the possibility of maintaining the scheduled time of treatment |
| Knop et al. 2014 [2]  Primary prophylaxis | Pegfilgrastim 6 mg day 6 | Phase 2  Newly diagnosed MM | 190 | Pegfilgrastim  Regimen: lenalidomide, doxorubicin and dexamethasone |  | No treatment-related mortality occurred during lenalidomide, doxorubicin and dexamethasone induction  Non-relapse mortality at 1 year from allogeneic SCT, 10.6%  Incidences of AEs  Pneumonia, 11%  VTE, 7.2%  Febrile neutropenia, 5.3% | Our data show lenalidomide, doxorubicin and dexamethasone induction to be very effective in patients with newly diagnosed MM and to be well tolerated. By subsequent double SCT, a large number of CRs/stringent CRs were added |
| Leleu et al. 2016 [3]  Reactive | Pegfilgrastim (dose NR) | Prospective, observational  RRMM  Proportion with moderate renal impairment  Pegfilgrastim, 38%  Daily filgrastim, 31% | 198 | Lenalidomide, with and without dexamethasone and G-CSF (filgrastim or pegfilgrastim) | Pegfilgrastim administered in 16 (8%)  12 patients received long courses of daily G-CSF; they had a greater reduction in lenalidomide exposure than the study population as a whole | No adverse drug reactions to G-CSF were reported | Long courses of daily G-CSF were observed in a subset of patients. The authors propose that these patients may be good candidates for management with pegfilgrastim |
| Radic Kristo et al. 2014 [4]  Primary prophylaxis | Pegfilgrastim 6 mg s.c. day +1 | Retrospective  MM | 94^a^ | Pegfilgrastim  (*n* = 51)  Filgrastim  (*n* = 68)  Administered with melphalan  Patients underwent ASCT | Median (range) time until leukocyte recovery, days  Pegfilgrastim, 10 (8–19)  Filgrastim, 11 (9–25)  *P =* NS  Median duration of febrile neutropenia, days  Pegfilgrastim, 2  Filgrastim, 2  *P =* NS  Mean/median (range) duration of hospitalization, days  Pegfilgrastim, 14.06/13 (11–23)  Filgrastim, 14.88/14  (6–29)  Total cost for GF after ASCT was not significantly different | NR | There were no significant differences between filgrastim and pegfilgrastim in the acceleration of leukocyte count recovery after ASCT, prevention of febrile neutropenia after ASCT or costs for GFs. Considering these results, the conclusions were that the main difference between G-CSF and the pegylated form was that a single fixed dose is more convenient than multiple daily administrations |
| Scott et al. 2014 [5]  Primary prophylaxis | Pegfilgrastim 6 mg day 6 | Phase 1 dose escalation  RRMM | 15 | Pegfilgrastim  Administered concomitantly with hydroxychloroquine (at ascending doses), cyclophosphamide and rapamycin |  | Reasons for study discontinuation  PD, 5  Lack of response, 6  Treatment toxicity, 2  Two dose-limiting toxicities:  diarrhoea (grade 3) related to hydroxychloroquine and thrombocytopenia (grade 4) possibly related to hydroxychloroquine, cyclophosphamide or rapamycin  Other significant AEs  *Grade 4*  Thrombocytopenia, 4  Neutropenia, 1  Lymphopenia, 5  *Grade 3*  Catheter-associated MRSA bacteraemia, 2  Diarrhoea, 2  Constipation, 2  Abdominal discomfort, 1  Prolonged QTc, 1  Three discontinued after one cycle owing to PD, toxicity and patient choice | The addition of mTOR and autophagy inhibition to a backbone of cyclophosphamide and dexamethasone yields a tolerable regimen with durable response |
| SCT mobilization | | | | | | | |
| Bailiff et al. 2013 [6] | Pegfilgrastim day +1  (dose NR) | Retrospective  Myeloma | 113 | Pegfilgrastim (*n* = 21)  Lenograstim (*n* = 52)  No G-CSF (*n* = 40)  Administered concomitantly with  high-dose melphalan  on day −1  Patients underwent ASCT | Median time for neutrophil engraftment, days  Pegfilgrastim, 12  Lenograstim, 12.74  *P =* 0.1078  Median inpatient stay, days  Pegfilgrastim, 16  Lenograstim, 16  *P =* 0.2112  Median days of i.v. broad-spectrum antibiotic use  Pegfilgrastim, 4.51  Lenograstim, 3.90  *P =* 0.4150  No difference in PFS or OS between the three cohorts (log-rank,  *P =* 0.455) | NR | The use of daily lenograstim from day +7 is an effective strategy in patients with MM undergoing ASCT. It is equally as effective as pegfilgrastim in reducing the time to neutrophil engraftment, duration of inpatient stay and antibiotic use. Both daily lenograstim from day +7 and pegfilgrastim are superior to no G-CSF use |
| Bouko et al. 2013 [7] | Pegfilgrastim  12 mg or 18 mg | Phase 2 RCT  Newly diagnosed MM | 68 | Filgrastim (*n* = 23)  Pegfilgrastim 12 mg  (*n* = 23)  Pegfilgrastim 18 mg  (*n* = 22)  Chemotherapy regimen NR | Time to CD34+ peak after GF administration, hours  Filgrastim, 72  Pegfilgrastim 12 mg, 96  Pegfilgrastim 18 mg, 96  Number of patients considered ‘mobilization failure’  Filgrastim, 1  Pegfilgrastim 12 mg, 1  Pegfilgrastim 18 mg, 2 | NR | The use of pegfilgrastim 18 mg showed no additional benefit |
| Costa et al. 2013 [8] | Pegfilgrastim 6 mg SC day +1 | Phase 1 dose escalation  Relapsed symptomatic MM | 12 | Pegfilgrastim  Administered concomitantly with melphalan and carfilzomib (both at ascending doses)  Patients underwent ASCT | Neutrophil engraftment  Median (range), 11 (8–15) days  Platelet engraftment  Median (range), 17.5 (11–24) days | There were no non-haematological grade 4 toxicities  Most frequent grade 3 toxicities (*N* = 12)  Infection, 7 (58.3%)  Pneumonia, 1 (8.3%) Bacteraemia, 1 (8.3%)  UTI, 1 (8.3%)  Febrile neutropenia, 4 (33.3%) | Conditioning with carfilzomib and melphalan before ASCT is well tolerated in patients with relapsed MM |
| Matar et al. 2015 [9] | Pegfilgrastim 6 mg or 12 mg day 4 | Retrospective  MM undergoing first immobilization | 119 | Daily apheresis with pegfilgrastim 6 mg  (*n* = 62) or pegfilgrastim 12 mg (*n* = 57) | CD34+ apheresis yield  8 × 10^6^ vs 7.4 × 10^6^  (*P =* 0.2)  CD34+ target (6 × 10^6^) reached  85.4% vs 73.7%  (*P =* 0.1)  Time to ANC engraftment  15 days for both groups  Time to platelet engraftment  21 days for both groups  Number of apheresis days  1.8 vs 1.7 days (*P =* 0.3) | No major complications or drug toxicities were identified | The use of pegfilgrastim 6 mg for autologous haematopoietic cell mobilization in patients with MM is more cost-effective and yields similar results to pegfilgrastim 12 mg |
| Mayer et al. 2015 [10] | Pegfilgrastim | Retrospective  MM | 142 | Induction treatment followed by stem cell mobilization with  G-CSF alone or by cyclophosphamide (3 g/m^2^) + G-CSF schedule  ASCT (*n* = 113); ASCT + high-dose melphalan (*n* = 29)  Pegfilgrastim day +1 post high-dose therapy (*n* = 22), lenograstim day +7 post-high-dose therapy (*n* = 84), or no G-CSF^b^ (*n* = 34) | Median duration for neutrophil engraftment  12 vs 12.7 vs 14 days  (*P =* 0.0005)  Median duration for platelet engraftment  21.5 vs 16.5 days, respectively, in the pegfilgrastim and no G-CSF groups (*P =* 0.253) | Not reported | The use of colony-stimulating factors (pegfilgrastim and lenograstim) is associated with a shorter time to neutrophil engraftment and reduced inpatient stay, which does not translate into PFS or OS advantage |
| De Mel et al. 2015 [11] | Pegfilgrastim 6 mg on day 4  (G-CSF 10 μg/kg/day from day 4 or 5 onwards in some patients) | Retrospective  MM | 133 | Vinorelbine and cyclophosphamide  with G-CSF or pegfilgrastim  (*n* = 84)  Cyclophosphamide  with G-CSF  (*n* = 47) | Median CD34+/kg collection  Vinorelbine and cyclophosphamide, 8.4  Cyclophosphamide, 11.3 *P =* 0.009  Stem cell collection  >5 × 10^6^/kg  Vinorelbine and cyclophosphamide, 85%  Cyclophosphamide, 95% *P =* 0.07 | NR | Vinorelbine + cyclophosphamide appears superior to cyclophosphamide in terms of the time taken for an adequate peripheral CD34+ count and predictability of the day of harvest. The incidence of harvest- related complications was greater with cyclophosphamide alone than with vinorelbine and cyclophosphamide |
| Tuazon et al. 2014 [12] | Pegfilgrastim  12 mg | Retrospective  Myeloma | 53 | Pegfilgrastim + plerixafor  (*n* = 37)  Cyclophosphamide, pegfilgrastim + plerixafor  (*n* = 16)  Patients underwent auto-SCT | Median collection days  Pegfilgrastim + plerixafor, 1  Cyclophosphamide, pegfilgrastim + plerixafor, 2  *P =* 0.29  Patients achieving target CD34+ cell dose of 6.0 × 10^6^/kg  Pegfilgrastim + plerixafor, 91.9%  Cyclophosphamide, pegfilgrastim + plerixafor, 81.3%  *P =* 0.26 | In the cyclophosphamide group, 41% were hospitalized owing to complications, and thus only nine patients (59%) received the planned dose of plerixafor, compared with 100% in the non-cyclophosphamide group  There were no hospitalizations in the pegfilgrastim and plerixafor group due to toxicity | The preferred method to mobilize autologous stem cells should be with pegfilgrastim and planned plerixafor because it is able to achieve the prescribed cell dose and is associated with less toxicity and lower risk of hospitalization. This analysis suggests that per 100 patients, a total of 100 days of plasmapheresis could be avoided if all patients were mobilized with GFs alone |
| Studies of mixed populations reporting MM-specific data | | | | | | | |
| Bayer et al. 2013 [13] | Pegfilgrastim 12 mg | Retrospective  Non-Hodgkin lymphoma  (*n* = 4)  Hodgkin lymphoma  (*n* = 2)  MM (*n* = 7) | 13 | Ifosfamide, carboplatin and etoposide-based chemotherapy + pegfilgrastim, or cyclophosphamide followed by pegfilgrastim; plerixafor with or without filgrastim added based on CD34 count | 11 patients from the full population were able to complete stem cell mobilization successfully and achieve target yields  Two patients with MM did not meet the collection goal for two transplantations but did have sufficient yield for one  Median total CD34 yield  Full population,  6.9 × 10^6^/kg  Patients with MM,  10.59 × 10^6^/kg  Mean total MNC yield  MM patients,  8.71 × 10^8^/kg  Average time for neutrophil engraftment in full population, 11.46 days  Average time for platelet engraftment in full population, 19.91 days | There were no major complications and no unanticipated side effects | The combination of plerixafor and filgrastim can be safely and successfully administered to patients with inadequate peripheral blood pre-CD34 counts following chemotherapy-based mobilization regimens. This will prevent any delay in the transplantation process |
| Kim et al. 2015 [14] | 6 mg or 12 mg, between 24 hours and 6 days after chemotherapy | Systematic review and meta-analysis of nine studies (one RCT, eight non-RCTs)  Various haematological malignancies, including MM | 719 | Pegfilgrastim  Filgrastim  Additional chemotherapy varied between studies  Patients underwent auto-HSCT | Subgroup analysis of harvest yield between treatments  Patients with lymphoma, SDM −0.354; 95% CI −0.619 to −0.089;  *P =* 0.009  Patients with MM, SDM 0.014; 95% CI −0.414 to 0.442; *P =* 0.948  In a subgroup analysis by disease type, pegfilgrastim mobilization resulted in an earlier leukocyte recovery rate (*P =* 0.004) in the MM group than in the lymphoma/leukaemia group | The most frequently reported AE associated with filgrastim and pegfilgrastim mobilization was pain  MM population safety results Fruehauf et al. reported one patient (3.8%) with mild and reversible grade 1 thoracic pain in the pegfilgrastim group Tricot et al. described grade 3 or higher bone pain requiring potent analgesics such as codeine or morphine in 5% of patients receiving pegfilgrastim, although safety results for the filgrastim group were not presented | Pegfilgrastim may be a convenient alternative to filgrastim in chemo-cytokine mobilization of PBSCs for patients with MM or lymphoma undergoing auto-HSCT, with advantages including an earlier apheresis start, a reduction in apheresis procedures required and fewer cytokine applications. There may also be benefits in patients with MM regarding engraftment post-HSCT |
| Schade et al. 2013 [15] | Pegfilgrastim (or filgrastim; dose NR) | Retrospective  MM (*n* = 128)  Lymphoma  (*n* = 64) | 192 | GFs (either filgrastim or pegfilgrastim; *n* = 73)  GFs + plerixafor  (*n* = 119) | None of age, sex, underlying disease (MM or lymphoma), type of GF utilized (filgrastim or pegfilgrastim), PB-D34+ count, number of blood volumes processed or use of plerixafor was significantly associated with collection efficiency in a multivariate analysis | NR | There is no difference in collection efficiency between patients mobilized with GFs or GFs + plerixafor, indicating similar dynamics of intra-apheresis recruitment with both mobilization approaches |

*AE* adverse event, *ANC* absolute neutrophil count, *ASCT* autologous haematopoietic stem cell transplantation, *CI* confidence interval, *CR* complete response, *FISH* fluorescence *in situ* hybridisation, *G-CSF* granulocyte-colony stimulating factor, *GF* growth factor, *HSCT* haematopoietic stem cell transplantation, *i.v.* intravenous, *MM* multiple myeloma, *MNC* mononuclear cell, *MR* minimal response, *MRSA* methicillin-resistant *Staphylococcus aureus*, *MTOR* mechanistic target of rapamycin, NR not reported, *NS* not significant, *OS* overall survival, *PBSC* peripheral blood stem cell, *PD* disease progression, *PFS* progression-free survival, *PR* partial response, *QTc* corrected QT interval, *RCT* randomized controlled trial, *RRMM* relapsed and/or refractory multiple myeloma, *s.c.* subcutaneously, *SCT* stem cell transplantation, *SD* stable disease, *SDM* weighted standard difference in means, *VGPR* very good partial response, *VTE* venous thromboembolism

^a^119 procedures

^b^Some patients received varying durations of conventional G-CSF if there was no neutrophil engraftment by day +12

**References**

1. Cerchione C, Catalano L, Pareto AE, Picardi M, Pane F (2015) Pegfilgrastim in primary prophylaxis of febrile neutropenia during chemotherapy of relapsed and refractory multiple myeloma: a real-life experience. Support Care Cancer 23 (2):301–302. doi:10.1007/s00520-014-2490-y

2. Knop S, Langer C, Engelhardt M, Mugge LO, Reichle A, Rosler W, Bassermann F, Hertenstein B, Sturm I, Rollig C, Ostermann H, Schafer-Eckart K, Ringhoffer M, Gunther A, Junghanss C, Biersack H, Strifler S, Bachinger A, Einsele H, Bargou RC (2014) Response to lenalidomide, doxorubicin and dexamethasone (RAD) in newly diagnosed multiple myeloma is independent of cytogenetic risk and retained after double stem cell transplant. Blood 124:177.

3. Leleu X, Terpos E, García Sanz R, Cooney J, O'Gorman P, Minarik J, Greil R, Williams C, Gray D, Szabo Z (2016) An international, multicenter, prospective, observational study of neutropenia in patients being treated with lenalidomide + dexamethasone for relapsed or relapsed/refractory multiple myeloma (RR-MM) Am J Hematol 91:806–811.

4. Radic Kristo D, Zatezalo V, Jaksic B, Acamovic B, Planinc-Peraica A, Ostojic Kolonic S (2014) Comparison of peg-filgrastim versus filgrastim after autologus peripheral blood stem cell transplantation in patients with multiple myeloma. Transplant 49(Suppl. 1):S456.

5. Scott EC, Vogl DT, Reasor-Heard S, Floyd K, Medvedova E, Spurgeon SE, Gordon M, Kratz A, Siegel MB, Loriaux M, Trubowitz P, Smith SD, Liu SQ, Arora R, Stadtmauer EA, Amaravadi RK, Maziarz RT (2014) A phase I study of hydroxychloroquine with infusional cyclophosphamide, pulse dexamethasone and rapamycin in patients with relapsed or refractory multiple myeloma. Blood 124:3449.

6. Bailiff B, Murthy V, Bratby L, Lancashire J, Kishore B, Nikolousis E, Lovell R, Paneesha S (2013) Lenograstim from day +7 and pegfilgrastim are equally effective in reducing time to neutrophil engraftment, antibiotic use and inpatient stay in patients with myeloma undergoing autologous transplant. Haematologica 98:153–154.

7. Bouko Y, Garderet L, Triffet A, Lambermont M, Deweweire A, Doyen C, Ikhlef S, Van Riet I, Goblet V, Mayne D, Robin V (2013) Mobilization of patients with newly diagnosed myeloma undergoing ASCT : Are we ready to switch from G-CSF to PEG G-CSF? Clin Lymphoma Myeloma Leuk 13(Suppl. 1): S122 – 123.

8. Costa LJ, Landau H, Venkata JK, Kang Y, Koehne G, Chung DJ, Lendvai N, Bentz T, Giralt S (2013) Phase 1 trial of carfilzomib + high dose melphalan conditioning regimen prior to autologous hematopoietic stem cell transplantation (AHSCT) for relapsed multiple myeloma. Blood 122:3329.

9. Matar S, Dany M, Chhabra S, Costa LJ, Stuart RK (2015) Pegfilgrastim 6 mg versus 12 mg for autologous stem cell mobilization in multiple myeloma patients: efficacy, safety, and cost analysis. Blood 126:4306.

10. Mayer G, Kishore B, Lovell R, Nikolouisis E, Paneesha S (2015) Colony stimulating factors (pegfilgrastim or lenograstim) use in patients with myeloma undergoing autologous peripheral blood stem cell transplantation (APBSCT) is associated with early neutrophil engraftment and shorter inpatient stay with no impact on progression free survival (PFS) or overall survival (OS). Blood 126:5450.

11. De Mel S, Chen Y, Lin A, Yap ES, Soh TG, Mah J, Ooi MG, Gopalakrishnan SK, Donato LKS, Halim NAA, Chng WJ, Tan LK (2015) Vinorelbine-cyclophosphamide compared to cyclophosphamide in peripheral blood stem cell mobilization for multiple myeloma. Blood 126:3103.

12. Tuazon S, Sharma M, Zhan T, Kasner M, Alpdogan O, Martinez U, Grosso D, Filicko J, Pro B, Wagner JL, Carabasi M, Flomenberg N, Weiss M (2014) Autologous stem cell mobilization with pegfilgrastim and planned plerixafor is equally effective and safer as compared with cyclophosphamide, pegfilgrastim and plerixafor. Biol Blood Marrow Transplant 20(2 Suppl.):S119. http://dx.doi.org/10.1016/j.bbmt.2013.12.175

13. Bayer RL, Keyzner A, Donahue L, Elera C, Roddy R (2013) Safety and efficacy of plerixafor (mozobil) addition to chemotherapy and growth factor stem cell mobilization regimens in patients with low pre-CD34 counts undergoing stem cell collection prior to autologous stem cell transplantation. Biol Blood Marrow Transplant 19(2 Suppl.):S179–S180. http://dx.doi.org/10.1016/j.bbmt.2012.11.170

14. Kim MG, Han N, Lee EK, Kim T (2015) Pegfilgrastim vs filgrastim in PBSC mobilization for autologous hematopoietic SCT: a systematic review and meta-analysis. Bone Marrow Transplantation 50 (4):523–530. doi:<http://dx.doi.org/10.1038/bmt.2014.297>

15. Schade H, Kang Y, Saurabh C, Stuart RK, Edwards KH, Kramer C, Schneider M, Littleton A, Shoptaw KB, Costa LJ (2013) Similar dynamics of intra apheresis autologous CD34+ recruitment and collection efficiency in patients undergoing mobilization with or without plerixafor. Blood 122:904.
